# Pyruvate kinase is a dosage-dependent regulator of cellular amino acid homeostasis

**DOI:** 10.18632/oncotarget.730

**Published:** 2012-11-01

**Authors:** Katharina Bluemlein, Matthias Glückmann, Nana-Maria Grüning, René Feichtinger, Antje Krüger, Mirjam Wamelink, Hans Lehrach, Stephen Tate, Daniel Neureiter, Barbara Kofler, Markus Ralser

**Affiliations:** ^1^ Department of Biochemistry & Cambridge Systems Biology Centre, University of Cambridge, Cambridge, United Kingdom; ^2^ AB SCIEX, Landwehrstrasse, Darmstadt, Germany; ^3^ Max Planck Institute for Molecular Genetics, Berlin, Germany; ^4^ Research Program for Receptor Biochemistry and Tumor Metabolism, Department of Pediatrics, Paracelsus Medical University, University Hospital Salzburg, Salzburg, Austria; ^5^ AB SCIEX, Concord, Ontario, Canada; ^6^ VU University Medical Center Amsterdam, Amsterdam, The Netherlands; ^7^ Institute of Pathology, Paracelsus Medical University, University Hospital Salzburg, Salzburg, Austria

**Keywords:** cancer metabolism, pyruvate kinase, proteomics, amino acid profile

## Abstract

The glycolytic enzyme pyruvate kinase (PK) is required for cancer development, and has been implicated in the metabolic transition from oxidative to fermentative metabolism, the Warburg effect. However, the global metabolic response that follows changes in PK activity is not yet fully understood. Using shotgun proteomics, we identified 31 yeast proteins that were regulated in a PK-dependent manner. Selective reaction monitoring confirmed that their expression was dependent on PK isoform, level and activity. Most of the PK targets were amino acid metabolizing enzymes or factors of protein translation, indicating that PK plays a global regulatory role in biosynthethic amino acid metabolism. Indeed, we found strongly altered amino acid profiles when PK levels were changed. Low PK levels increased the cellular glutamine and glutamate concentrations, but decreased the levels of seven amino acids including serine and histidine. To test for evolutionary conservation of this PK function, we quantified orthologues of the identified PK targets in thyroid follicular adenoma, a tumor characterized by high PK levels and low respiratory activity. Aminopeptidase AAP-1 and serine hydroxymethyltransferase SHMT1 both showed PKM2- concentration dependence, and were upregulated in the tumor. Thus, PK expression levels and activity were important for maintaining cellular amino acid homeostasis. Mediating between energy production, ROS clearance and amino acid biosynthesis, PK thus plays a central regulatory role in the metabolism of proliferating cells.

## INTRODUCTION

Cancer cell growth is characterized by re-configuration of cellular metabolism. Different routes of intermediary and biosynthetic metabolism, including nucleic acid synthesis, amino acid biosynthesis, and energy production are reconfigured [1-4]. A characteristic feature of many tumors is an augmentation of non-oxidative (fermentative) metabolism at the expense of oxidative metabolism (respiration), although the latter would be more efficient for energy production. Known as the ‘Warburg effect’, this metabolic reconfiguration is considered as a ‘hallmark’ of cancer, and may have high therapeutic potential [5, 6].

Recent results indicate that the Warburg effect could be the result of metabolic balancing processes which avoid the accumulation of toxic reactive oxygen species (ROS) maintaining metabolic homeostasis [7, 8]. Indeed, cancer cells suffer from high loads of ROS which may make them vulnerable in pro-oxidant therapies [8-10]. ROS originate from biochemical reactions, i.e. the degradation of fatty acids, and leakage from complex I and III of the respiratory chain [11, 12]. Clearing of these reactive molecules prevents oxidative stress, and is conducted by large machinery which involves central carbon metabolism as essential metabolic component. Fluxes of glycolysis and the adjacent pentose phosphate pathway (PPP) are adjusted when cells encounter an oxidative environment [13]. This metabolic reconfiguration fuels the antioxidative machinery with its main redox cofactor NADPH, required by the glutathione, peroxiredoxin and thioredoxin systems [14, 15], and is involved in regulating gene expression during oxidative conditions [16].

The metabolic enzyme pyruvate kinase (PK) is required for coordination of these pathways when cells encounter increased ROS levels. Lowering PK activity in yeast and human increases resistance to oxidants [17, 18]. At least in part, this adaptation is mediated through accumulation of the PK substrate phosphoenolpyruvate. PEP causes feedback inhibition of several glycolytic enzymes, including triosephosphate isomerase (TPI) [17]. Lowered TPI activity increases oxidant resistance as studied in yeast and *C. elegans*, and acts through re-directing the metabolic flux from glycolysis into the PPP. This increases the NADP^+^ reduction rate [13], and triggers an adaptation of gene expression towards oxidative conditions [16].

In this study, we focused on the regulatory events upstream and downstream of PK, picturing PK induced proteome changes in yeast and human. For this, we combined workflows of quantitative proteomics which circumvent the use of isotope labeled standards (‘label-free proteomics’). In yeast, low levels of PK were sufficient to induce changes in the biosynthetic proteome, primarily affecting protein biosynthesis and amino acid metabolism. Subsequently, ion exchange chromatography was used to profile free amino acid levels in yeast strains possessing different PK activities. Low PK activity caused a decline in the concentration of arginine, aspartic acid, histidine, lysine, serine, threonine, and valine, and an increase in glutamine and glutamate, thus triggered a broad reconfiguration of the yeast amino acid profile.

In human thyroid follicular adenoma, we observed upregulation of two orthologues of the identified yeast proteins, aminopeptidase AAP-1 and serine hydroxymethyltransferase SHMT1, and provide evidence that their expression level might quantitatively correlate with PKM2 expression in the cancer state. Thus, in yeast and humans, PK emerges as regulator of carbohydrate, energy and also amino acid metabolism.

## RESULTS

### PK levels target cellular biosynthetic metabolism

Although of reduced catalytic activity [18, 19], the expression level of the predominant human pyruvate kinase isoform PKM2 is increased in cancers [20]. In yeast, changes in PK isoform and expression levels are observed when cells switch from fermentation to oxidative metabolism [17, 21], indicting that PK plays an evolutionary conserved role in the underlining metabolic reconfiguration.

Previously, we have shown that lowering PK activity in yeast increases the rate of respiration, and at the same time augments oxidant resistance [17]. This indicates this enzyme is required for coordination of respiration and ROS clearance. Here, we used mass spectrometry based proteomics to picture the global physiological consequences triggered by a change in PK levels. We compared two engineered yeast strains that just differ in the promoter (*TEF1_pr_* for high level expression and *CYC1_pr_* for low level expression) used to express yeast Pyk1p, the high active yeast PK isoform [17, 21]. MS/MS shotgun proteome profiles of these two yeast strains were recorded using a nanoLC (Ultimate 3000 (Dionex)) coupled QqTOF mass spectrometer TripleTOF5600 (AB/Sciex) [22]. Obtained MS/MS spectra were used to identify peptides and proteins by a database search with ProteinPilot™ software (AB SCIEX). A typical run yielded the identification of 922 proteins at global FDR of <1% from the UniprotKB/Swiss-Prot database 57.15. At a global FDR of 1% on the peptide level 6393 peptides and on spectrum level 72256 spectra were identified.

**Figure 1:**
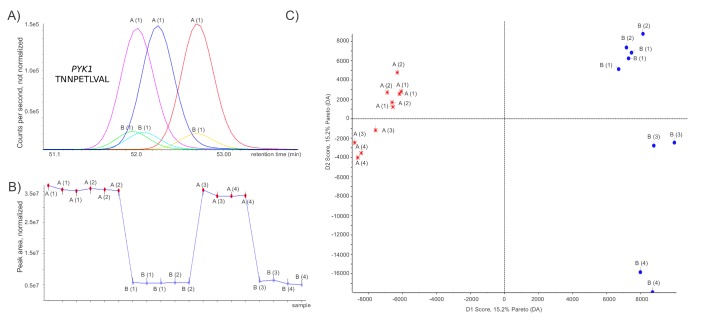
Label-free shotgun proteome profiles of yeast expressing Pyk1p at high and low level (A)Tryptic digests generated from yeast protein extracts were analyzed on an AB SCIEX QqTOF mass spectrometer. Extracted ion chromatograms for the Pyk1p tryptic peptide *K.TNNPETLVAL.R* from three LC-MS/MS shotgun runs each of yeast expressing Pyk1p at high level (‚A‘ samples) or low level (‚B‘ samples). (B)Peak area of all Pyk1p-associated peptides in cells expressing Pyk1p at and high and low level, normalized to the total peak area of the respective run. Replicate injections of the same sample (LC-MS technical replicates) are marked with the same label; Numbers (1) & (2), respectively (3) & (4) indicate sample preparations and tryptic digests from independently grown yeast cultures. (C)Principal component analysis after pareto scaling of the shotgun proteome profiles, generated with MarkerView (AB/Sciex). A change in the Pyk1p expression level clearly separates the proteomes. Determined by two tailed T-test, 31 proteins (Table 1) were significantly (p < 0.05) regulated and responsible for the separation of the proteome profiles.

The identified proteins and peptides were analyzed with PeakView™ software (AB SCIEX), where mass and extracted ion chromatograms (XICs) were obtained for the confidentially identified peptides (Peptide ID with unique peptides at a global false discovery rate < 1%). The same peptides were extracted for all technical and biological replicates. The extraction window for all peptide XICs was set to 0.01 Da to increase specificity and improve the quantification results. Typical elution profiles for peptides show a width of 20 seconds (FWHM) at the HPLC conditions selected to ensure a number of > 8 points for quantification, typically at <35 points across peak base. Extracted ion chromatograms of the tryptic Pyk1p peptide K.TNNPETLVAL.R of cells with different PYK1 expression levels, 19 data points per peak, illustrate clear separation between high (A) and low (B) level expression of PK in three replicates each (Fig 1A). For relative quantification, sum of the total peak area was used to normalize peak areas between the different samples. Fig 1B illustrates the normalized peak area for all identified Pyk1p-belonging peptides over technical and biological replicates. In total, 42.6% of identified proteins met the stringent criteria of being identified with > 3 unique peptides in all 19 data-dependent acquisition runs, thus were used for quantification.

### PK targets respond to level changes of both yeast PYK isozymes

We first analyzed the shotgun proteome profiles by principal component analysis (PCA) after pareto sorting. Profiles obtained from both from technical and biological replicates showed clear clustering according to whether Pyk1 was expressed at high or low level (Fig 1C). Thus, a change in the expression level of the Pyk1p enzyme caused a reproducible change in the yeast proteome.

**Figure 2:**
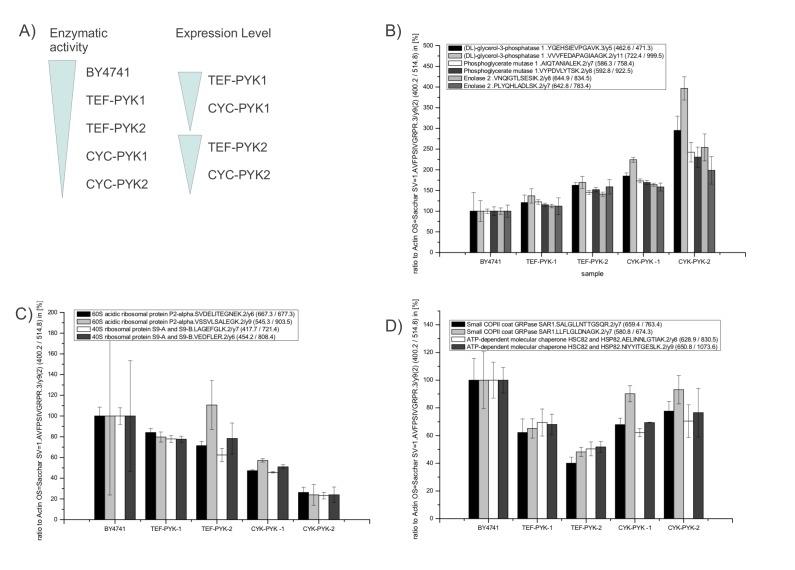
Targeted quantification of PK regulated genes by selected reaction monitoring (A)Overview of the yeast model strains [17] that differ in overall PYK activity, Pyk isoform, and expression level. (Left bars) Total PK activity changes over BY4741 > *TEFpr-PYK1 > TEFpr-PYK2 > CYCpr-PYK1* to *CYCpr-PYK2*, as described previously [17]. (Right bars). High expression of Pyk1p and Pyk2p under control of the *TEF1* promoter, low expression under control of the CYC1 promoter [17]. (B)Glycolytic enzymes glycerol-3-phosphatase, phosphoglycerate mutase 1, and enolase 2 are upregulated in yeast strains with lower PK activity. (C)Ribosomal proteins quantified using single reaction monitoring (60S acidic ribosomal protein P2-alpha and 40S ribosomal protein S9-A and S9-B) are downregulated in yeast strains with lower PK activity. (D)PYK targets Sar1 and Hsp82 are regulated in strains with altered PYK1 expression, but are not correlating with the overall enzyme activity. (A-D) Determined expression values are given in the Suppl. Table 1.

We continued our investigations by an in depth verification of the proteomic results making use of a quantitative MS technique, selective reaction monitoring (SRM). In difference to shotgun proteomics, SRM assays are generated in a targeted way and on the base of the protein/peptide sequence or its known fragmentation spectrum [23]. With SRM, a much lower number of molecules per sample can be quantified (here we choose nine proteins randomly), but these assays are more sensitive due to improved signal to noise ratio, therefore facilitating faster runtimes, and are more reproducible, and are thus used in diagnostics [23-25]. We established and validated SRM assays for a representative number of proteins which were significantly (p < 0.05) responsible for separating the proteomes in the PCA (Fig 1C, Table 1). The SRM assays were applied to BY4741, the two Pyk1p strains used in the QqTOF analysis (Fig 1A), and two further yeast strains (*TEF_pr_-Pyk2 and CYC_pr_-Pyk2*) which express the low active Pyk2 isozyme at high and low level (please see Fig 2A for an overview, strains were in detail described in [17])

**Table 1:** 

Upregulated (TEFpr-PYK1 compared to CYCpr-PYK1)						
Row				t-value	p-value	Fold Change
1	P00549	KPYK1_YEAST		72.27618	1.33E-22	6.287671
2	P00815	HIS2_YEAST		15.3411	2.17E-11	2.445479
3	P09436	SYIC_YEAST		13.50707	1.61E-10	1.729696
4	P22943	HSP12_YEAST		12.0193	9.82E-10	1.674836
5	P37898	AAP1_YEAST		9.55664	3.00E-08	4.608135
8	P05750	RS3_YEAST		8.541646	1.48E-07	1.249022
10	P15108	HSC82_YEAST		8.178904	2.70E-07	1.291876
12	Q07478	SUB2_YEAST		7.612269	7.13E-07	1.446553
13	P05694	METE_YEAST		7.49884	8.71E-07	1.289915
16	P20606	SAR1_YEAST		6.706529	3.69E-06	4.203254
17	P05319	RLA2_YEAST		5.961237	1.55E-05	1.204707
19	P05755	RS9B_YEAST		5.801303	2.13E-05	1.172176
22	P37292	GLYM_YEAST		5.658496	2.83E-05	1.264169
23	P31539	HS104_YEAST		5.619102	3.07E-05	1.279804
25	P19358	METK2_YEAST		5.567696	3.40E-05	1.388585
26	P19882	HSP60_YEAST		5.533043	3.65E-05	1.177094
27	P49089	ASNS1_YEAST		5.473253	4.12E-05	1.361167
28	P38011	GBLP_YEAST		5.441745	4.40E-05	1.325665
29	P48164	RS7B_YEAST		5.351547	5.29E-05	1.221251
30	P41940	MPG1_YEAST		5.328021	5.55E-05	1.420641
32	P27476	NSR1_YEAST		5.180612	7.52E-05	1.511833
**Downregulated (TEFpr-PYK1 compared to CYCpr-PYK1)**						
**Row**			**t-value**		**p-value**	**Fold Change**
6	P00924	ENO1_YEAST	-9.00757		7.00E-08	0.577695
7	P38720	6PGD1_YEAST	-8.83961		9.14E-08	0.70217
9	P00358	G3P2_YEAST	-8.21728		2.53E-07	0.403518
11	Q00055	GPD1_YEAST	-7.84349		4.77E-07	0.689863
14	P00830	ATPB_YEAST	-7.24239		1.38E-06	0.789716
15	P41277	GPP1_YEAST	-6.71021		3.66E-06	0.664699
18	P00950	PMG1_YEAST	-5.90746		1.72E-05	0.757085
20	Q05911	PUR8_YEAST	-5.76513		2.29E-05	0.720522
21	Q07551	KAR_YEAST	-5.71729		2.52E-05	0.652231
24	P38972	PUR4_YEAST	-5.61894		3.07E-05	0.823931
31	P00925	ENO2_YEAST	-5.21596		6.99E-05	0.828283

Label-free SRM confirmed the PYK-dependent regulation as identified with the shotgun platform for 7 of the 9 proteins (Fig 2, Suppl. Table 1 for numeric values). Interestingly, all Pyk1p regulated proteins were also dependent on the Pyk2p expression level, thus they were not regulated in an isoform specific manner. Remarkably, we observed that the expression pattern grouped the PK targets according to their biological function. The first group of co-regulated proteins was formed by the glycolytic enzymes glycerol-3-phosphatase dehydrogenase, phosphoglycerate mutase, and enolase 2. All these proteins were gradually upregulated as lower overall activity of PK was (Fig 2B). Second, ribosomal proteins grouped together, and showed the exact opposite trend being down-regulated in strains with lower PK activity (Fig 2C). Finally, there was the third group of proteins which was clearly affected by altered PYK expression, but did not correlate with the enzymatic activity (Fig 2D). This group was represented by *SAR1*, a small COPII coat GTPase and HSC82/ HSP82, an ATP-dependent molecular chaperone. These proteins were expressed at quite similar levels in *TEF-PYK1* and *TEF-PYK2* yeast, at higher levels in *CYC-PYK1* and *CYC-PYK2* yeast, and even higher levels in the wild-type strain BY4741(Fig 2D).

Hence, low PK activity led to an up-regulation of glycolytic proteins assayed, and at the same time to a downregulation of tested components of the translational machinery. Furthermore, the proteome study identified PK target proteins which responded in an activity independent manner. Finally, all proteins found to be regulated by *PYK1* were also controlled by *PYK2*, Thus, both expression level and enzymatic activity of PK show to have an effect on protein expression regulation.

### PK expression levels affect the concentration of free amino acids

**Figure 3:**
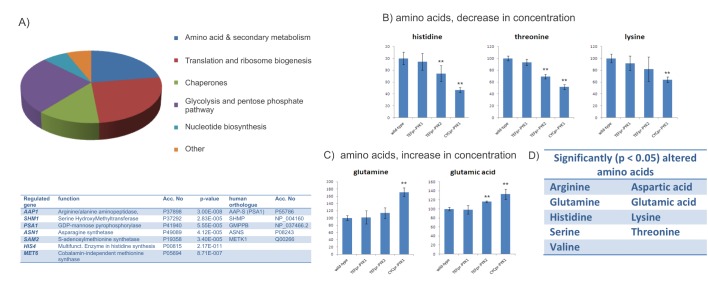
Amino acid and biosynthethic metabolism are targets of yeast PK (A)PK targets identified in by proteome profiling belong to primary metabolism as well to amino acid- and protein biosynthesis (upper panel). The table lists the genes which play a role in amino acid and protein biosynthethic metabolism, and their direct human orthologue (if applicable) (B)Pyruvate kinase affects the concentration of nine amino acids. Illustrated is the concentration of histidine, threonine and lysine (decreased) or (C)Glutamine, glutamate (increased) relative to their level in BY4741 (wild-type). Significant (p<0.05, two-tailed T test) concentration changes are marked (**). (D)In total, nine out of 17 amino acids were found to be significantly regulated (p< 0.05, two-tailed T test), by PK activity; these amino acids except glutamine and glutamate were present in lower concentrations in yeast with low PK activity.

Virtually all proteins responsible for the clear division of the PYK proteomes were involved in energy, biosynthetic and intermediary metabolism as well as translation (including ribosome biogenesis and protein folding) (Fig 3A). This indicates that changing PK levels triggered metabolic reactions and pathways required for the biosynthesis of proteins (biosynthethic metabolism). Importantly, the list of significantly regulated yeast genes contained several enzymes involved in the synthesis of amino acids. Amino acid concentrations are central for biosynthetic metabolism, important for growth control, and at the same time implicated in cancer metabolism [1, 26, 27]. Therefore, we continued our survey by quantifying 17 amino acid levels using ninhydrin detection/ion exchange chromatography in wild-type as well as the yeast cells with altered Pyk1p and Pyk2 expression levels. Indeed, PK expression had large effects on the amino acid profiles, nine of the 17 profiled amino acids were significantly (P < 0.05) altered by a change in the PK expression level (Fig 3). Histidine, threonine or lysine, for instance were strongly decreased in cells with low PYK activity (Fig 3B), whereas glutamine and glutamate were detected at higher concentration (Fig 3C). Only eight amino acids, including methionine, glycine or valine did not significantly respond to a change in the activity of PK (Suppl. Table 2). Thus, PK levels influence the expression of proteins involved in biosynthetic metabolism, and cause a strong signature in the amino acid profile.

### PKM2- dependent regulation of AAP-S and SHMP in Thyroid Follicular Adenoma

Both, the enzymatic/ glycolytic functions of PK, and their role in the regulation of redox processes are highly conserved from yeast to humans [7, 8]. Therefore we speculated that also this newly discovered aspect of the PK function could be conserved. Therefore, we selected PK targets for which we could identify a human orthologue, and tested if they were expressed at sufficient level to be quantified by SRM in thyroid follicular adenoma.

**Figure 4:**
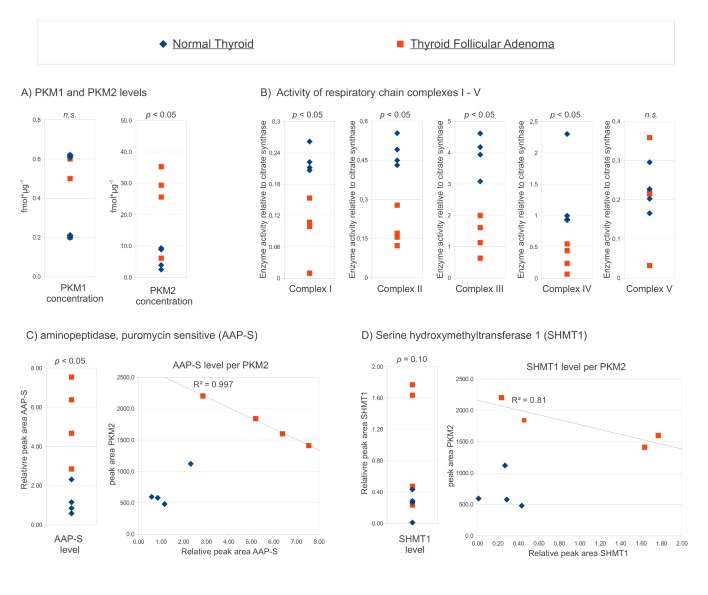
AAP-S and SHMT1 are upregulated in thyroid follicular adenoma and correlate with the PKM2 expression level (A)PKM2, but not PKM1 concentration, distinguishes thyroid follicular adenoma from normal thyroid. PKM1 and PKM2 were quantified in tumor and the controls [20], the absolute PKM concentrations in individual biopsies are plotted. (B)Activity of the respiratory chain is reduced in thyroid follicular adenoma. Enzyme activity of complexes I – V of the four tumors and control tissues relative to the activity of citrate synthase. (C)Puromycin sensitive aminopeptidase (AAP-S) is upregulated in thyroid follicular adenoma, and correlates with PKM2 in the tumor state. Relative level of AAP-S in tumor and normal thyroid (left panel), AAP-S expression level plotted versus the PKM2 concentration in the same biopsy (right panel). (D)Serine hydroxymethyltransferase 1 (SHMT1) shows clear trends towards higher expression in thyroid follicular adenoma, and seem to correlate with PKM2 in the tumor state. Relative level of SHMT1in tumor and normal thyroid (left panel), SHMT1expression level plotted versus the PKM2 concentration (right panel). (A-D) *p- values* were determined with two-tailed T test*, n.s*.: non significant

Thyroid follicular adenomas are benign human cancers which exhibit clear up-regulation of *PKM2* when compared to normal thyroid tissue [20] (Fig 4A for a summary). First, we determined the activity of the five respiratory chain complexes in these samples. The enzymatic activities were normalized to the citrate synthase, which is a marker of mitochondrial mass. A clear reduction in respiratory chain activity (complexes I – IV) was observed in thyroid follicular adenomas compared to normal adjacent thyroid tissue (Fig 4 B). Thus, exhibiting upregulation of PKM2, and low activity of the respiratory chain, follicular thyroid adenomas represented an archetypal tissue exhibiting the Warburg effect.

Using the QTRAP mass spectrometer, we tested if human orthologues of the yeast proteins can be quantified by label free LC-SRM in the trypsin digested cell extracts [25]. We could identify peptides and SRM transitions suitable for robust relative quantification of the aminopeptidase AAP-S and serine hydroxymethyltransferase SHMT1 (Fig 4). Specific SRM signals corresponding to the other three proteins were of too low signal or specificity for a reliable quantification with our method (data not shown). In adenoma, both AAP-S and SHMT1 were present in higher concentration compared to normal thyroid tissue (Fig 4C), showing that these proteins are upregulated in the tumor state. The effect was prominent for AAP-S; the AAP-S levels clearly distinguished normal and adenoma tissue (p < 0.01). Next, we plotted the relative expression level of AAP-S versus the concentration of *PKM2* (Fig 4C, right panel) Both, AAP-S and SHMT1 did not correlate with the expression level of PKM2 in the healthy tissue. However, both proteins showed correlation with the PKM2 level in the tumor state (Fig 4C). AAP-S levels exhibited an almost perfect linear correlation with the PKM2 concentration. Thus, direct orthologues of two yeast PK target genes were upregulated in human thyroid follicular adenomas. Moreover, their expression correlates with the PKM2 level in the tumor, but not in healthy tissue.

## DISCUSSION

Reaction cascades of central metabolic pathways are well characterized, yet still relatively little is known about their regulation and coordination. However, homeostasis of cellular metabolites and metabolic fluxes is essential for cellular growth, and needs to be adapted to the growth conditions of all living cells [3, 28-32] Recently, it has become clear that the metabolic enzyme PK is required for proper coordination of energy- and redox metabolism. In its glycolytic role, PK converts phosphoenolpyruvate (PEP) to pyruvate, yielding a molecule of ATP, and thus, this reaction is required for the net ATP production in glycolysis [33]. Interest in PKM2 had increased markedly after it had been suggested that PKM2 expression in adults could be specific to cancer cells, and that a PKM1 to PKM2 isoform switch might be responsible for increased fermentative metabolism (Warburg effect) of cancer cells [34, 35]. Cancer specificity of PKM2 could however not be confirmed in recent studies, as PKM2 was found to dominate over PKM1 in most adult tissue, including kidney, lung, liver, thyroid, bladder and colon [20], except however muscle [34]. Despite not specific to cancer, several studies established an essential role of PKM2 in metabolic regulation and the oxidative stress response, processes of high importance for cancer development [17-19, 36-40]. First, the literature appears consistent that PKM2 is upregulated on transcript or protein level in several malignant and benign tumors. These studies included human melanoma [41] childhood leukemia [42], renal cell carcinoma, bladder carcinoma, hepatocellular carcinoma, colorectal carcinoma, lung carcinoma, thyroid follicular adenoma [20], and mouse kidney tumors [36]. Several cancer tissues also expressed the PKM1 isoform, however at much lower levels than PKM2. In some but not all tumors, also the PKM1 isoform was found to be upregulated [20].

Conversely, at the same time it has been reported that the PK activity is reduced by posttranslational modifications in cancer cells, and that this reduction is required for their proliferation [18, 19, 43]. Reduced PK activity was attributed to PKM2 phosphorylation [19], thiol oxidation [18], and acetylation [43]. This indicates that PKM2 is upregulated in cancer to increase the protein content, but not to increase the overall PK enzymatic activity. However, it has also to be noted that it is yet unclear to which extent the identified modifications might also be found on PKM2 in normal human tissue, and thus, and as to which extend they are cancer cell specific.

Here, we followed the proteome wide consequences of the Pyk1p downregulation by creating shotgun proteome of yeast expressing Pyk1p at high and low levels using a latest-generation QqTOF proteomics platform, a TripleTOF™ 5600 (AB SCIEX) mass spectrometer coupled to a high-pressure nanoLC system was used for a label free proteome profiling. Using the paragon research engine, we identified >900 proteins per single run. A set of 393 proteins met the stringent criteria for being quantified, i.e. that a minimum of three specific peptides were identities in all 19 shotgun runs. The proteome profiles clearly clustered dependent on the PK expression level (Fig 1C) This clear separation was caused by 7.8 percent (31) proteins that were significantly responding to changing PK levels.

These numbers illustrate both the advantages and disadvantages of current label free proteomic approaches. On the plus side, label-free profiling is much more flexible and convenient, as isotope labeled standards are costly, and these standards can only be created for a limited number of species. In this particular case, i.e. SILAC labeling was difficult, as the yeast genetic modifications necessary [44] would impact yeast amino acid transport and metabolism, and thus potentially interfere with the biological function of PK. On the down side, at the current state of the art, the number of proteins quantifiable in a labeled experiments is higher, as the peptide pools can easily be pre-separated before the LC runs. However, also when using isotope labels, the number of replicate injections may be limited by the throughput of the nanoLC gradients (i.e. the 19 injections using at a 3hr gradient required one week of instrument time). These current bottlenecks for label-free approaches might however improve in the near future, as recent developments in data-independent acquisition (i.e. SWATH [45]) will facilitate significant faster runtime while increasing in the number of quantifiable peptides and proteins.

To verify the proteome profiles, we applied the targeted MS technique selective reaction monitoring (SRM) and analyzed the expression of nine randomly chosen PYK targets. Facilitated by the shorter runtimes and the high reproducibility of the SRM experiments, we included three additional strains that have different PK activities, the wild type parent BY4741, as well as two yeast strains expressing the *PYK2* both at high and low level [17]. For seven of the nine tested proteins, this extended study did confirm Pyk1p, and also Pyk2p dependent regulation. Furthermore, the SRM experiments allowed grouping of these PK targets according to their co-expression pattern. Remarkably, co-expressed proteins formed functional clusters: proteins up-regulated were glycolytic enzymes, proteins downregulated were involved in translation. PK dependence of glycolytic enzyme expression is an interesting parallel to the humans in respect to cancer development: hydroxylated *PKM2* leads to an upregulation of glycolytic enzymes through interacting with hypoxia inducible factor -1α (HIF1α) [38, 39]. Thus, in both species regulation of glycolytic enzyme abundances can be attributed to PK, and hence, at least also parts of non-catabolic functions of PK appear to be conserved between yeast and humans. Interestingly, we also observed overlapping targets upon an activation of the PPP during the oxidative stress response [16]. As low PYK activity increases the concentration of PPP metabolites [17], it is conceivable that the PPP is involved in gene expression regulation mediated by PK. In this context is has to be noted that switches of glycolysis to the PPP can howeveronly partly attributed to transcriptional regulation, i.e. metabolic shifts in glycolysis and the PPP can be regulated on the metabolic level only [46], and may occur much faster than transcriptional regulation [47].

The vast majority of identified PK targets were involved in metabolic processes related to glycolysis, the synthesis of nucleic acids, amino acids and proteins. Although it is assumed that PK mediates the regulation of glycolysis [39, 40], similar mechanisms might also exist for amino acid metabolism. To obtain a metabolic picture of the global PK function in this process we thus set out to use ion exchange chromatography and ninhydrin detection to profile the levels of free amino acids in the PK model strains. Comparing yeast with different PK expression level and activity, we found a clear reconfiguration of the overall amino acid profile. In cells with low PK activity, seven of 17 amino acids were significantly less concentrated, and two were higher concentrated.

Amino acid levels are important indicators for the metabolic homeostasis of cells – and closely interconnected with protein metabolism. In yeast, mammalian cells, and flies, proteasome inhibition is lethal due by causing amino acid shortage [48], implying that maintaining sufficient amino acid supply is crucial for proliferating cells and tumors. One of the amino acids we found at decreased concentration was serine. Remarkably, it has recently been described for human cells that PKM2 stimulates de novo serine biosynthesis [49], and that in turn serine can act as allosteric activator of PKM2 [50]. This implies a feedback circle involving PYK that controls serine production in mammalian cells, providing evidence that the PK's role in regulation of serine metabolism is conserved between yeast and mammals.

Only two amino acids increased in concentration when there was low PYK activity, glutamine and glutamate. Turnover of these two amino acids may be of crucial importance for cancer metabolism, as cells can obtain a substantial amount of their energy through the process of glutaminolysis [3, 51]. A change in pyruvate kinase expression seem thus shift energy metabolism towards glutaminolysis, providing sufficient energy supply for cancer cells.

To test for conservation in humans, we searched for human orthologues of the proteins regulated by yeast PK. Remarkably, two direct orthologues were known to be associated with cancer progression. The first protein, asparagine synthase, is the orthologue of yeast protein ASN1, ASNS (Fig 3). ASNS is as potential target for the treatment of castration-resistant stage of prostate cancer [52]. Moreover, the expression level of this enzyme is an indicator for the efficiency of the enzyme drug L-Asparaginase (L-ASP) in ovarian cancer [53]. The second protein, serine hydroxymethylstransferase (SHMT1), is the human orthologue of the yeast protein *SHM1*. SHMT1 catalyzes the formation of glycine and methylene tetrahydrofolate which are important precursors for nucleotide biosynthesis, and is discussed as a therapeutic target since the late 1980s [54].

Taken together, these observations implicated that the function of PK in regulating amino acid metabolism is conserved between yeast and human. Therefore, we continued our investigations by quantifying further potential PK targets in a human tumor. For two reasons, we selected thyroid follicular adenoma for these investigations: First, these benign cancers are characterized by a clear upregulation of the *PKM2* enzyme [20]. Indeed, normal and adenoma biopsies are clearly distinguished by the *PKM2* level ([20], graphically illustrated in Fig 4A). Also *PKM1* was expressed in these tumor tissues, but at around a 50 fold lower level. In difference to PKM2, *PKM1* concentrations did not distinguish healthy thyroid from follicular adenoma tissue. Second, as adenomas are slow growing tumors, one can exclude unspecific effects which may result from altered metabolism of the rapidly proliferating cells. Finally, the activity of the respiratory chain complexes I – IV, determined in both the healthy as well as the adenoma biopsies, indicated significantly lowered activity of mitochondrial metabolism (Fig 4B). Thus, being characterized by increased *PKM2* levels and a decrease in respiratory chain efficiency, thyroid follicular adenoma represented tissue exhibiting classic features of the ‘Warburg effect’

SRM assays were tested for all five human proteins, but only SHMT1 and the aminopeptidase AAP-S were detected at sufficient intensity for reliable relative quantification by our LC-MS/MS workflow. Remarkably, AAP-S and SHMT1 were both found clearly upregulated in these adenoma samples. At the measure of the relative expression level, AAP-S levels clearly distinguished adenoma and that of control tissue (p < 0.01). Plotted against the absolute concentration of PKM2, both targets created clusters that distinguished the adenomas from the control tissue. Thus, expression of two direct protein orthologues to the yeast PK targets correlate with PKM2 expression in thyroid follicular adenoma.

## CONCLUSIONS

Pyruvate kinase emerges as a central hub in the regulation of metabolic pathways important for cell proliferation and cancer. As a glycolytic enzyme, it is responsible for the net ATP yield of glycolysis, and the control of the redox state. Here, a combined analysis of label-free shotgun proteomics, targeted proteomics and amino acid profiling reveals that this protein is further important for the coordination of biosynthesis of an orchestra of amino acids. Glutamine and glutamate levels were highly increased in cells with low PK expression levels, which seem to connect the process of glutaminolysis with the regulation of carbon metabolism. Seven of the remaining amino acids were decreased in concentration, including serine, which has recently been reported to be a direct regulator of PKM2 activity in humans [49, 50]. Finally, studies in thyroid follicular adenoma indicated that part of this PK function is conserved in man, and revealed two protein biomarkers of this disease state. AAP-S and SHMT1 correlated with the PKM2 expression level and were upregulated in the tumor. Thus, PK plays an evolutionary conserved role in the coordination of carbon and amino acid homeostasis, making it and its downstream effectors promising candidates for modulating (cancer) metabolism for therapeutic purposes.

## METHODS

### Yeast strains and growth conditions

*Δpyk1Δpyk2* yeast strains expressing Pyk1p or Pyk2p under control of the *TEF1* or *CYC1* promoter were described previously [17]. The *Δpyk2* strain was generated from BY4741 by single gene replacement with kanMX4, and verified by PCR and SRM. Yeast was grown in yeast peptone (YP) media containing 2% dextrose, 2% galactose, 3% ethanol/ 0.1% glucose or 2% raffinose as indicated. All measurements were performed on mid-log batch cultures.

### Enzyme assays

Follicular thyroid adenoma and normal adjacent thyroid tissues (20-100 mg) were homogenized with a tissue disintegrator (Ultraturrax, IKA, Staufen, Germany) in extraction buffer (20 mM Tris-HCl, pH 7.6, 250 mM sucrose, 40 mM KCl, 2 mM EGTA) and finally homogenized with a motor-driven Teflon-glass homogenizer (Potter S, Braun, Melsungen, Germany). The homogenate was centrifuged at 600*g* for 10 min at 4°C. The supernatant (600*g* homogenate) containing the mitochondrial fraction was used for measurement of enzyme activities. OXPHOS enzyme and citrate synthase activity [55-59] were measured as described earlier. Activity of respiratory chain complexes is given relative to citrate synthase activity.

### Shotgun proteomics

An TripleTOF™ 5600 system (AB SCIEX, Concord, Ontario, Canada) was coupled with a Dionex Ultimate 3000 RSLCnano system (Dionex, Sunnyvale, California). Details to instrumental parameters and data processing can be found in the supplementary material. Briefly the raw data was analyzed using ProteinPilot™ Software v.4.0 (AB SCIEX) on database UniprotKB/Swiss-Prot 57.15 in thorough search mode applying the integrated false discovery rate analysis tool (PSPEP) [60].

Subsequent quantification of peptides and proteins was performed automatically in PeakView™ Software for proteins with <1% false discovery rate as performed using PSPEP. Areas of peptides and proteins were automatically transferred to MarkerView™ Software for visualization of differences for technical replicates and statistical analysis using principal component analysis followed by principal component variable grouping to identify protein groups with similar trends in regulation.

### Selective reaction monitoring

(SRM) analysis was performed on a QTRAP^®^ 5500 hybrid triple quadrupole/ ion trap mass spectrometer instrument (AB SCIEX, Concord, ON, Canada) coupled to an Eksigent 2D ultra nano LC system (Eksigent, CA, USA) as described earlier [20, 25]. In brief, SRM assay development for the target proteins was developed using the MRMPilot™ software (AB SCIEX) version 2.1. Identity of peptides was confirmed using the MIDAS™ workflow (MRM Initiated Detection And Sequencing) [25, 61]. Quantification of the potential PYK targets was achieved by normalization to cellular reference proteins [25] V-type proton ATPase catalytic subunit A (V-ATPase) and actin. The ratios of their SRM intensities were similar in all samples investigated. SRM transitions and collision energy (CE) settings are given in the Suppl. Table 3.

### Amino acid Analysis

Amino acid analysis was performed using ion-exchange chromatography on a Biochrom 30 amino acid analyzer with ninhydrin detection. Each sample was analyzed in triplicate. 200 μl sample was added to 200 μl of 0.5 mM DL-2,4-diamino-n-butyratedihydrochloride (used as internal standard) and 4.9 % 5-sulfosalicylic acid to precipitate the protein. Samples were mixed and centrifuged for 10 minutes at 3345g and the supernatant was filtered (Ultrafree-MC Durapore PVDF, 0.22 μm; Waters, UK) for 10 minutes at 3345g to remove all protein precipitate. A series of lithium citrate buffer solutions were run through a lithium column (8 μm Biochrom, UK) containing the samples. Individual amino acids were eluted according to their pH. Reaction with ninhydrin was utilized to elicit a spectrum of colors at different wavelengths giving a chromatogram representing the concentrations of each amino acid.

## ETHICS

Four thyroid follicular adenomas and adjacent normal appearing thyroid tissue from patients were obtained from the Institute of Pathology, University Hospital Salzburg, Austria.

The study was performed according to the Austrian Gene Technology Act. Experiments were performed in accordance with the Helsinki declaration of 1975 (revised 2000) and the guidelines of the Salzburg State Ethics Research Committee as not being a clinical drug trial nor epidemiological investigation. Furthermore, the study did not extend to examination of individual case records. The anonymity of the patients has been ensured.

## Supplementary Tables and Text




