# Supplementary material for: Pyruvate kinase is a dosage-dependent regulator of cellular amino acid homeostasis
Source: Oncotarget. 2012 Nov 1;3(11):1356–69. doi: 10.18632/oncotarget.730 (PMC3717798; doi:10.18632/oncotarget.730)
Supplement: Supplementary file 1 [file oncotarget-03-1356-s001.docx]

**Pyruvate kinase is a dosage-dependent regulator of cellular amino acid homeostasis-Bluemlein et al**

**Supplementary Table1: Data supplement for Figure 2**

|  |  |  |  | |  | | |  | | |  |  | |  | |
| --- | --- | --- | --- | --- | --- | --- | --- | --- | --- | --- | --- | --- | --- | --- | --- |
| **Heat shock SV=1.LIDEYGDDFAK.2/y9 (643.3 / 1059.4)** |  |  |  | |  | | |  | | |  |  | |  | |
|  |  |  |  | |  | | |  | | |  |  | |  | |
|  | MV | STDEV | **MV [%]** | | **STDEV [%]** | | | **corrected STDEV** | | | |  | |  | |
| BY4741 | 0.11 | 0.01058 | **100** | | 9.912117 | | |  | | |  |  | |  | |
| TEF-PYK-1 | 0.10 | 0.00454 | **97.49672** | | 4.257091 | | | 10.83123 | | |  |  | |  | |
| TEF-PYK-2 | 0.09 | 0.00855 | **86.30616** | | 8.008962 | | | 10.25565 | | |  |  | |  | |
| CYK-PYK -1 | 0.08 | 0.00429 | **76.80805** | | 4.01938 | | | 10.65352 | | |  |  | |  | |
| CYK-PYK-2 | 0.08 | 0.00824 | **78.38334** | | 7.71861 | | | 11.15137 | | |  |  | |  | |
|  |  |  |  | |  | | |  | | |  |  | |  | |
|  | | | |  | |  |  | |  |  | | |  | |  |
| **Heat shock SV=1.LIDEYGDDFAK.2/y9 (643.3 / 1059.4)** |  |  |  | |  | | |  | | | | |  |  |  |
|  |  |  |  | |  | | |  | | | | |  |  |  |
|  | MV | STDEV | **MV [%]** | | **STDEV [%]** | | | **corrected STDEV** | | | | | |  |  |
| BY4741 | 0.03 | 0.00288 | **100** | | 9.868413 | | |  | | | | |  |  |  |
| TEF-PYK-1 | 0.03 | 0.00308 | **98.15054** | | 10.55109 | | | 14.59267 | | | | |  |  |  |
| TEF-PYK-2 | 0.03 | 0.00192 | **97.03355** | | 6.593009 | | | 12.71717 | | | | |  |  |  |
| CYK-PYK -1 | 0.03 | 0.00219 | **89.49208** | | 7.519094 | | | 10.80552 | | | | |  |  |  |
| CYK-PYK-2 | 0.03 | 0.00394 | **86.36692** | | 13.52013 | | | 17.76654 | | | | |  |  |  |

| **Adenylosuc lyase SV=1.DVNNALQPFQK.2/y6 (637.3 / 760.4)** |  |  |  |  |  |  |
| --- | --- | --- | --- | --- | --- | --- |
|  |  |  |  |  |  |  |
|  | **MV** | **STDEV** | **MV [%]** | **STDEV [%]** | **corrected STDEV** | |
| BY4741 | 0.05 | 0.00540 | **100** | 10.86125 |  |  |
| TEF-PYK-1 | 0.06 | 0.00337 | **121.724** | 6.766568 | 12.20117 |  |
| TEF-PYK-2 | 0.06 | 0.00086 | **114.3967** | 1.730488 | 5.761086 |  |
| CYK-PYK -1 | 0.05 | 0.00091 | **95.3257** | 1.824358 | 2.439462 |  |
| CYK-PYK-2 | 0.05 | 0.01006 | **98.64277** | 20.21996 | 20.58731 |  |

| **Adenylosuc lyase SV=1.VTELLGFDK.2/y6 (511.3 / 692.4)** |  |  |  |  |  |  |
| --- | --- | --- | --- | --- | --- | --- |
|  |  |  |  |  |  |  |
|  | **MV** | **STDEV** | **MV [%]** | **STDEV [%]** | **corrected STDEV** | |
| BY4741 | 0.03 | 0.01951 | **100** | 60.8338 |  |  |
| TEF-PYK-1 | 0.04 | 0.00088 | **115.6607** | 2.749283 | 60.88022 |  |
| TEF-PYK-2 | 0.05 | 0.00754 | **151.1674** | 23.49821 | 15.72519 |  |
| CYK-PYK -1 | 0.04 | 0.00086 | **111.6898** | 2.68784 | 15.72967 |  |
| CYK-PYK-2 | 0.03 | 0.01300 | **105.2365** | 40.539 | 38.5969 |  |

| **(DL)-glyce 1 SV=3.YGEHSIEVPGAVK.3/y5 (462.6 / 471.3)** |  |  |  |  |  |  |  |
| --- | --- | --- | --- | --- | --- | --- | --- |
|  |  |  |  |  |  |  |  |
|  | **MV** | **STDEV** | **MV [%]** | **STDEV [%]** | **corrected STDEV** | |  |
| BY4741 | 1.23 | 0.55507 | **100** | 44.98357 |  |  |  |
| TEF-PYK-1 | 1.49 | 0.22727 | **120.6062** | 18.41828 | 47.50513 |  |  |
| TEF-PYK-2 | 2.00 | 0.08311 | **162.4869** | 6.735393 | 15.824 |  |  |
| CYK-PYK -1 | 2.28 | 0.09336 | **184.993** | 7.565863 | 5.823157 |  |  |
| CYK-PYK-2 | 3.64 | 0.43114 | **294.9207** | 34.93967 | 12.5332 |  |  |

| **(DL)-glyce 1 SV=3.VVVFEDAPAGIAAGK.2/y11 (722.4 / 999.5)** |  |  |  |  |  |  |  |
| --- | --- | --- | --- | --- | --- | --- | --- |
|  |  |  |  |  |  |  |  |
|  | **MV** | **STDEV** | **MV [%]** | **STDEV [%]** | **corrected STDEV** | |  |
| BY4741 | 0.19 | 0.04875 | **100** | 25.33706 |  |  |  |
| TEF-PYK-1 | 0.26 | 0.03320 | **136.978** | 17.253 | 28.29509 |  |  |
| TEF-PYK-2 | 0.33 | 0.02823 | **169.6085** | 14.6684 | 15.27874 |  |  |
| CYK-PYK -1 | 0.43 | 0.01143 | **224.2572** | 5.93816 | 9.044671 |  |  |
| CYK-PYK-2 | 0.76 | 0.05369 | **396.5502** | 27.90243 | 7.518039 |  |  |

| **Phosphoglycerate mutase SV=3.AIQTANIALEK.2/y7 (586.3 / 758.4)** |  |  |  |  |  |  |
| --- | --- | --- | --- | --- | --- | --- |
|  |  |  |  |  |  |  |
|  | **MV** | **STDEV** | **MV [%]** | **STDEV [%]** | **corrected STDEV** | |
| BY4741 | 1.04 | 0.05755 | **100** | 5.552683 |  |  |
| TEF-PYK-1 | 1.27 | 0.05772 | **122.3738** | 5.568907 | 7.179239 |  |
| TEF-PYK-2 | 1.51 | 0.04958 | **145.6925** | 4.783242 | 5.611415 |  |
| CYK-PYK -1 | 1.80 | 0.04783 | **173.2735** | 4.614609 | 4.227458 |  |
| CYK-PYK-2 | 2.51 | 0.24458 | **242.4129** | 23.59693 | 10.09193 |  |

| **Phosphogly mutase SV=3.VYPDVLYTSK.2/y8 (592.8 / 922.5)** |  |  |  |  |  |  |
| --- | --- | --- | --- | --- | --- | --- |
|  |  |  |  |  |  |  |
|  | **MV** | **STDEV** | **MV [%]** | **STDEV [%]** | **corrected STDEV** | |
| BY4741 | 1.88 | 0.20081 | **100** | 10.67414 |  |  |
| TEF-PYK-1 | 2.17 | 0.07169 | **115.5687** | 3.810914 | 11.17189 |  |
| TEF-PYK-2 | 2.86 | 0.09714 | **151.8131** | 5.163623 | 4.73736 |  |
| CYK-PYK -1 | 3.18 | 0.09398 | **169.1905** | 4.995671 | 4.504136 |  |
| CYK-PYK-2 | 4.34 | 0.46073 | **230.4656** | 24.49068 | 11.0292 |  |

| **Enolase 2 SV=2.VNQIGTLSESIK.2/y8 (644.9 / 834.5)** |  |  |  |  |  |  |
| --- | --- | --- | --- | --- | --- | --- |
|  |  |  |  |  |  |  |
|  | **MV** | **STDEV** | **MV [%]** | **STDEV [%]** | **corrected STDEV** | |
| BY4741 | 9.19 | 0.71749 | **100** | 7.803192 |  |  |
| TEF-PYK-1 | 10.35 | 0.39031 | **112.5111** | 4.244939 | 8.667446 |  |
| TEF-PYK-2 | 13.00 | 0.45894 | **141.3819** | 4.991279 | 5.167031 |  |
| CYK-PYK -1 | 15.08 | 0.34445 | **163.9722** | 3.746205 | 4.205123 |  |
| CYK-PYK-2 | 23.36 | 2.99709 | **254.0626** | 32.59557 | 13.03157 |  |

| **Enolase 2 SV=2.PLYQHLADLSK.2/y7 (642.8 / 783.4)** |  |  |  |  |  |
| --- | --- | --- | --- | --- | --- |
|  |  |  |  |  |  |
|  | **MV** | **STDEV** | **MV [%]** | **STDEV [%]** | **corrected STDEV** |
| BY4741 | 0.33 | 0.04853 | **100** | 14.61218 |  |
| TEF-PYK-1 | 0.37 | 0.06697 | **112.0742** | 20.16653 | 23.17966 |
| TEF-PYK-2 | 0.53 | 0.05804 | **158.774** | 17.47791 | 21.09402 |
| CYK-PYK -1 | 0.53 | 0.03173 | **158.4882** | 9.553102 | 12.55027 |
| CYK-PYK-2 | 0.66 | 0.11019 | **198.5974** | 33.1803 | 17.76139 |

| **Small COPII SV=1.SALGLLNTTGSQR.2/y7 (659.4 / 763.4)** |  |  |  |  |  |  | |  |
| --- | --- | --- | --- | --- | --- | --- | --- | --- |
|  |  |  |  |  |  |  | |  |
|  | **MV** | **STDEV** | **MV [%]** | **STDEV [%]** | **corrected STDEV** | | |  |
| BY4741 | 0.09 | 0.01366 | **100** | 15.82208 |  | |  |  |
| TEF-PYK-1 | 0.05 | 0.00847 | **62.16769** | 9.811098 | 22.34724 | |  |  |
| TEF-PYK-2 | 0.03 | 0.00391 | **39.98761** | 4.525651 | 19.42035 | |  |  |
| CYK-PYK -1 | 0.06 | 0.00395 | **67.89998** | 4.573014 | 13.16997 | |  |  |
| CYK-PYK-2 | 0.07 | 0.00601 | **77.61526** | 6.957942 | 11.21268 | |  |  |

| **Small COPII SV=1.LLFLGLDNAGK.2/y7 (580.8 / 674.3)** |  |  |  |  |  |
| --- | --- | --- | --- | --- | --- |
|  |  |  |  |  |  |
|  | **MV** | **STDEV** | **MV [%]** | **STDEV [%]** | **corrected STDEV** |
| BY4741 | 0.05 | 0.01069 | **100** | 20.54287 |  |
| TEF-PYK-1 | 0.03 | 0.00361 | **65.10996** | 6.947574 | 23.14886 |
| TEF-PYK-2 | 0.03 | 0.00172 | **48.20363** | 3.305633 | 12.68414 |
| CYK-PYK -1 | 0.05 | 0.00303 | **90.22582** | 5.816842 | 9.412271 |
| CYK-PYK-2 | 0.05 | 0.00531 | **93.12002** | 10.21199 | 12.72113 |

| **ATP-depend molecular SV=4.AELINNLGTIAK.2/y8 (628.9 / 830.5)** |  |  |  |  |  |
| --- | --- | --- | --- | --- | --- |
|  |  |  |  |  |  |
|  | **MV** | **STDEV** | **MV [%]** | **STDEV [%]** | **corrected STDEV** |
| BY4741 | 0.70 | 0.09099 | **100** | 12.99203 |  |
| TEF-PYK-1 | 0.49 | 0.06836 | **69.42647** | 9.761374 | 19.14359 |
| TEF-PYK-2 | 0.35 | 0.03525 | **50.37952** | 5.032986 | 17.24781 |
| CYK-PYK -1 | 0.43 | 0.02045 | **62.10815** | 2.920226 | 11.0413 |
| CYK-PYK-2 | 0.49 | 0.08238 | **70.48263** | 11.76368 | 17.33982 |

| **ATP-depend molecular SV=4.NIYYITGESLK.2/y9 (650.8 / 1073.6)** |  |  |  |  |  |
| --- | --- | --- | --- | --- | --- |
|  |  |  |  |  |  |
|  | **MV** | **STDEV** | **MV [%]** | **STDEV [%]** | **corrected STDEV** |
| BY4741 | 0.23 | 0.02091 | **100** | 9.128439 |  |
| TEF-PYK-1 | 0.16 | 0.01705 | **68.04809** | 7.443063 | 14.24665 |
| TEF-PYK-2 | 0.12 | 0.00925 | **51.82193** | 4.035956 | 13.42734 |
| CYK-PYK -1 | 0.16 | 0.00062 | **69.38473** | 0.269571 | 7.797809 |
| CYK-PYK-2 | 0.18 | 0.04021 | **76.51862** | 17.5495 | 22.93823 |

**Suppl. Table 2: Amino acid levels in BY4741 and yeast strains with low PK activity.** Values are given relative to the concentration of
BY4741. n=3 for each measurement, Stdev : Standard deviation, TTest, 2 tailed TTest

|  | BY4741 | Stdev | *TEF_pr_-PYK1* | Stdev | *TEF_pr_-PYK2* | Stdev | *CYC_pr_-PYK1* | Stdev | *p* |
| --- | --- | --- | --- | --- | --- | --- | --- | --- | --- |
| *increased* |  |  |  |  |  |  |  |  |  |
| glutamine | 100.00 | 6.82 | 101.89 | 18.29 | 113.51 | 14.23 | 171.09 | 11.90 | ** |
| glutamic acid | 100.00 | 3.67 | 98.12 | 9.22 | 116.09 | 1.82 | 132.68 | 11.33 | ** |
|  |  |  |  |  |  |  |  |  |  |
| *decreased* |  |  |  |  |  |  |  |  |  |
| histidine | 100.00 | 10.42 | 94.34 | 14.23 | 74.34 | 13.24 | 46.59 | 4.14 | ** |
| threonine | 100.00 | 4.21 | 93.23 | 5.66 | 69.21 | 3.76 | 51.69 | 3.97 | ** |
| arginine | 100.00 | 8.71 | 103.97 | 8.63 | 89.46 | 15.57 | 55.14 | 3.16 | ** |
| lysine | 100.00 | 7.11 | 91.75 | 12.12 | 81.97 | 20.74 | 64.02 | 4.51 | ** |
| aspartic acid | 100.00 | 6.50 | 94.74 | 10.78 | 82.14 | 5.12 | 84.31 | 2.60 | ** |
| serine | 100.00 | 7.75 | 92.11 | 5.09 | 84.63 | 4.62 | 77.03 | 8.65 | ** |
|  |  |  |  |  |  |  |  |  |  |
|  |  |  |  |  |  |  |  |  |  |
| *not significant* |  |  |  |  |  |  |  |  |  |
| alanine | 100.00 | 15.79 | 91.36 | 12.53 | 103.09 | 2.03 | 113.37 | 8.77 |  |
|  |  |  |  |  |  |  |  |  |  |
| leucine | 100.00 | 18.83 | 89.96 | 9.76 | 85.80 | 12.97 | 80.52 | 5.21 |  |
| phenylalanine | 100.00 | 16.40 | 92.48 | 9.19 | 86.66 | 13.15 | 83.74 | 6.95 |  |
| glycine | 100.00 | 15.38 | 93.30 | 11.35 | 103.15 | 6.60 | 88.14 | 7.66 |  |
| tyrosine | 100.00 | 27.56 | 84.78 | 9.48 | 85.87 | 15.33 | 76.09 | 7.61 |  |
| isoleucine | 100.00 | 19.82 | 87.08 | 9.31 | 83.19 | 10.07 | 70.28 | 3.87 |  |
| methionine | 100.00 | 14.08 | 92.41 | 10.96 | 88.62 | 11.44 | 92.76 | 9.94 |  |
| valine | 100.00 | 20.11 | 93.36 | 13.07 | 109.78 | 10.10 | 93.60 | 3.97 |  |
|  |  |  |  |  |  | ** = *CYC_pr_-PYK1* to BY4741: p < 0.05 | | | |

**Supplementary Table 3: MRM transitions and collision energy.**

| protein | tryptic peptide | Q1 | Q3 | CE |
| --- | --- | --- | --- | --- |
| *Saccharomyces cerevisiae* |  |  |  |  |
| (DL)-glycerol-3-phosphatase 1 | YGEHSIEVPGAVK | 462.6 (3/y5) | 471.3 (3/y5) | 22 |
| (DL)-glycerol-3-phosphatase 1 | VVVFEDAPAGIAAGK | 722.4 (2/y11) | 999.5 (2/y11) | 37 |
| Phosphoglycerate mutase 1 | AIQTANIALEK | 586.3 (2/y7) | 758.4 (2/y7) | 31 |
| Phosphoglycerate mutase 1 | VYPDVLYTSK | 592.8 (2/y8) | 922.5 (2/y8) | 26 |
| Enolase 2 | VNQIGTLSESIK | 644.9 (2/y8) | 834.5 (2/y8) | 33 |
| Enolase 2 | PLYQHLADLSK | 642.8 (2/y7) | 783.4 (2/y7) | 38 |
| Small COPII coat GRPase SAR1 | SALGLLNTTGSQR | 659.4 (2/y7) | 763.4 (2/y7) | 34 |
| Small COPII coat GRPase SAR1 | LLFLGLDNAGK | 580.8 (2/y7) | 674.3 (2/y7) | 36 |
| ATP-dependent molecular chaperone HSC82 and HSP82 | AELINNLGTIAK | 628.9 (2/y8) | 830.5 (2/y8) | 33 |
| ATP-dependent molecular chaperone HSC82 and HSP82 | NIYYITGESLK | 650.8 (2/y9) | 1073.6 (2/y9) | 34 |
| 60S acidic ribosomal protein P2-alpha | SVDELITEGNEK | 667.3 (2/y6) | 677.3 (2/y6) | 34 |
| 60S acidic ribosomal protein P2-alpha | VSSVLSALEGK | 545.3 (2/y9) | 903.5 (2/y9) | 29 |
| 40S ribosomal protein S9-A and S9-B | LAGEFGLK | 417.7 (2/y7) | 721.4 (2/y7) | 23 |
| 40S ribosomal protein S9-A and S9-B | VEDFLER | 454.2 (2/y6) | 808.4 (2/y6) | 25 |
| Actin | AVFPSIVGRPR | 400.2 (3/y9) | 514.8 (3/y9) | 24 |
| V-type proton ATPase catalytic subunit A | VGHDNLVGEVIR | 436.6 (3/y5) | 573.3 (3/y5) | 21 |
|  |  |  |  |  |
| *Human* |  |  |  |  |
| P55786 ; yeast orthologue: AAP1 YHR047C | LSVEGFAVDK | 532.8 (2/y6) | 636.3 (2/y6) | 28 |
| P55786; yeast orthologue: AAP1 YHR047C | LNLGTVGFYR | 570.3 (2/y7) | 799.4 (2/y7) | 30 |
| NP_004160; yeast orthologue: SHMP | DSDVEVYNIIK | 647.8 (2/y6) | 749.5 (2/y6) | 34 |
| NP_004160; yeast orthologue: SHMP | ALSEALTELGYK | 647.8 (2/y7) | 823.5 (2/y7) | 34 |
