# Supplementary material for: Pyruvate kinase is a dosage-dependent regulator of cellular amino acid homeostasis
Source: Oncotarget. 2012 Nov 1;3(11):1356–69. doi: 10.18632/oncotarget.730 (PMC3717798; doi:10.18632/oncotarget.730)
Supplement: Supplementary file 2 [file oncotarget-03-1356-s002.docx]

SUPPLEMENTARY MATERIAL, MS and search settings

An TripleTOF™ 5600 system (AB SCIEX, Concord, Ontario, Canada, [1]) was coupled with a Dionex Ultimate 3000 RSLCnano system (Dionex, Sunnyvale, California).The solvents used were water with 0.1% (v/v) formic acid and solvent B comprising acetonitrile with 0.1% (v/v) formic acid. 10 μl of digest sample where loaded onto the precolumn (nano trap 75 μm x 2 cm, C18, 2 μm material, 100 Ǻ, Dionex) over 20 min at a flow rate of 5 μl/min. The material was eluted from the analytical column (nano column 75 μm x 15 cm, C18, 2 μm material, 100 Ǻ, Dionex) using a non linear gradient (One to 30% B in 50 min, 25 to 50% B in 20 min). The column was regenerated by washing at 95% solvent B for 5 minutes and re-equilibrated at 5% solvent B for 30 minutes.

For data dependent analysis experiments the TripleTOF™ 5600 system was operated using a 200 ms survey scan (TOF MS) followed by subsequent MS/MS interrogation of top 50 ions, MS resolution was up to <40000 while MS/MS resolution was maintained at <25000 full with at half maximum (FWHM) where each MS/MS event was recorded over 50 ms. Precursor ions with an intensity greater than 100 counts per second, charge state greater than 2^+^, and which were not present in dynamic exclusion list were selected and fragmented. After fragmentation the precursor was excluded for 10 sec. Ions were isolated using a quadrupole resolution of 0.7 Da and were fragmented in the collision cell using collision energy ramped from 15 eV up to 45 eV within the MS/MS accumulation time. In rare instances that less than the 50 precursors were detected the total cycle time was maintained at 2750 ms, allowing for longer scan times of the detected precursors in MS/MS mode.

Data Processing: From the LC MSMS raw data the peaklists were generated and analyzed using ProteinPilot™ Software v.4.0 (Revision Number: 146595) using the Paragon™ algorithm (Version 4.0.0.0, 146588) on 13118 proteins specific to the species Saccharomyces cerevisiae from database UniprotKB/Swiss-Prot 57.15 (download date March 3, 2010) in thorough search mode applying the integrated false discovery rate analysis tool. The false discovery rate (FDR) analysis invoked during a search with the Paragon™ algorithm is performed by a component of ProteinPilot™ software called the Proteomics System Performance Evaluation Pipeline (PSPEP) Software as described by [2]

The search parameters selected within ProteinPilot™ software were as follows: Identification of proteins, no Cystein alkylation under selection of the ID focus, allowing biological modifications. Digestion using enzyme trypsin selected, instrument TripleTOF™ 5600 allowing initial instrument specific MS tolerances of <0.05 Da and MSMS tolerances of <0.1 Da with a standard deviation of all MS values of <0.0011 Da and in MSMS mode <0.01 Da. No special search factors like phosphorylation emphasis or user modified parameter files were allowed for the search.

**References**

1. Andrews GL, Simons BL, Young JB, Hawkridge AM and Muddiman DC. Performance characteristics of a new hybrid quadrupole time-of-flight tandem mass spectrometer (TripleTOF 5600). Analytical chemistry. 2011; 83(13):5442-5446.

2. Tang WH, Shilov IV and Seymour SL. Nonlinear fitting method for determining local false discovery rates from decoy database searches. Journal of proteome research. 2008; 7(9):3661-3667.
